# Dandy-Walker malformation and Wisconsin syndrome: novel cases add further insight into the genotype-phenotype correlations of 3q23q25 deletions

**DOI:** 10.1186/1750-1172-8-75

**Published:** 2013-05-16

**Authors:** Alessandro Ferraris, Laura Bernardini, Vesna Sabolic Avramovska, Ginevra Zanni, Sara Loddo, Elena Sukarova-Angelovska, Valentina Parisi, Anna Capalbo, Stefano Tumini, Lorena Travaglini, Francesca Mancini, Filip Duma, Sabina Barresi, Antonio Novelli, Eugenio Mercuri, Luigi Tarani, Enrico Bertini, Bruno Dallapiccola, Enza Maria Valente

**Affiliations:** 1 Mendel Laboratory, IRCCS Casa Sollievo della Sofferenza, San Giovanni Rotondo, FG, Italy; 2 Department of Neurology, University Children’s Hospital, St Cirilus and Methodius University, Skopje, Macedonia; 3 Unit of Neuromuscular Disorders, Laboratory of Molecular Medicine, Bambino Gesù Pediatric Hospital IRCCS, Rome, Italy; 4 Department of Experimental Medicine, Sapienza University, Rome, Italy; 5 Department of Endocrinology and Genetics, University Children’s Hospital, St Cirilus and Methodius University, Skopje, Macedonia; 6 Department of Pediatric Endocrinology, G. D’Annunzio University, Chieti, Italy; 7 Child Neuropsychiatry Unit, Catholic University, Rome, Italy; 8 Department of Pediatrics and Child Neuropsychiatry, Sapienza University, Rome, Italy; 9 Department of Medical Genetics, Bambino Gesù Pediatric Hospital IRCCS, Rome, Italy; 10 Department of Medicine and Surgery, University of Salerno, Salerno, Italy

**Keywords:** Dandy-Walker malformation, Wisconsin syndrome, 3q deletion, *ZIC1-ZIC4* genes

## Abstract

**Background:**

The Dandy-Walker malformation (DWM) is one of the commonest congenital cerebellar defects, and can be associated with multiple congenital anomalies and chromosomal syndromes. The occurrence of overlapping 3q deletions including the *ZIC1* and *ZIC4* genes in few patients, along with data from mouse models, have implicated both genes in the pathogenesis of DWM.

**Methods and results:**

Using a SNP-array approach, we recently identified three novel patients carrying heterozygous 3q deletions encompassing *ZIC1* and *ZIC4*. Magnetic resonance imaging showed that only two had a typical DWM, while the third did not present any defect of the DWM spectrum. SNP-array analysis in further eleven children diagnosed with DWM failed to identify deletions of *ZIC1-ZIC4*. The clinical phenotype of the three 3q deleted patients included multiple congenital anomalies and peculiar facial appearance, related to the localization and extension of each deletion. In particular, phenotypes resulted from the variable combination of three recognizable patterns: DWM (with incomplete penetrance); blepharophimosis, ptosis, and epicanthus inversus syndrome; and Wisconsin syndrome (WS), recently mapped to 3q.

**Conclusions:**

Our data indicate that the 3q deletion is a rare defect associated with DWM, and suggest that the hemizygosity of *ZIC1-ZIC4* genes is neither necessary nor sufficient *per se* to cause this condition. Furthermore, based on a detailed comparison of clinical features and molecular data from 3q deleted patients, we propose clinical diagnostic criteria and refine the critical region for WS.

## Background

Dandy-Walker malformation (DWM, MIM%220200) represents one of the commonest (1:30,000 live births) congenital defects of cerebellar development, and a frequent cause of termination of fetuses diagnosed prenatally [1]. The key anatomical elements include hypoplasia and upward rotation of the cerebellar vermis associated to cystic dilatation of the fourth ventricle. This usually communicates with a retrocerebellar cyst, causing enlargement of the posterior fossa (PF) and elevation of the tentorium [2]. The severity of this malformation is highly variable, and conditions characterized by less hypoplasia, mild or absent vermis rotation, and mild cystic dilatation of the PF are part of the DWM spectrum. Hydrocephalus is found in 70-90% of patients, and in several cases DWM is part of a syndromic condition variably associated with heart, face and ocular abnormalities. Clinical presentation includes apnea episodes, hypotonia, seizures, cerebellar signs such as ataxia and nystagmus, spasticity, as well as macrocephaly and other features suggestive of hydrocephalus. Moderate to severe intellectual disability is common, although rare cases have been reported with normal intelligence, who were incidentally diagnosed in adult age [3,4].

DWM is usually a sporadic disorder and its genetic background remains poorly understood. Several cytogenetic abnormalities have been detected in syndromic cases, with involvement of large genomic regions that justify the multiorgan involvement [5]. In 2004, overlapping deletions including 3q24q25.1 chromosome region were reported in eight DWM patients displaying marked clinical and neuroradiological variability. Haploinsufficiency of *ZIC1* and *ZIC4* genes, mapping within the deleted region, has been implicated as causative of DWM, based on mouse models. Seven patients shared a 7 Mb critical region including both genes. In another patient, in which the deletion did not contain *ZIC1* and *ZIC4*, the expression level of both genes was found to be halved, suggesting a position effect [6]. Following this report, at least six additional cases with features of the DWM spectrum have been published, who carried a 3q chromosomal deletion encompassing the *ZIC1* and *ZIC4* genes [7-12].

Here we report on three novel patients with an interstitial deletion of 3q including both *ZIC1* and *ZIC4*, only two of which presented with DWM.

## Patients and methods

### Patients

Patients described in this study have been recruited as part of ongoing projects focused on the molecular characterization of cerebellar and brainstem congenital defects, and of intellectual disabilities. For each patient, a detailed clinical questionnaire and brain magnetic resonance imaging were obtained. Written informed consent was given by all families, and the study was approved by the Ethics Committee of Casa Sollievo della Sofferenza Hospital.

### SNP-Array analysis

GeneChip 6.0 platform (Affymetrix, Santa Clara, CA) was used to analyze patients’ genomic DNA following manufacturer’s instructions. Copy number analysis was performed with the Genotyping Console v4.0 (Affymetrix), and Copy Number Variations (CNV) were filtered as reported [13]. All CNV coordinates were mapped to the Genome Reference Consortium Human Build 37 (hg19), accessed from the UCSC genome browser (http://genome.ucsc.edu/)(accessed on May 22, 2013).

## Results

We performed CNV analysis by SNP-array in thirteen patients diagnosed with DWM of variable severity, including six isolated and seven syndromic cases. Two patients were found to carry heterozygous 3q deletions encompassing both *ZIC1* and *ZIC4* genes. Among the others, one patient showed a deletion of chromosome 8p [14], while the remaining cases resulted negative. We also detected a 3q deletion including *ZIC1* and *ZIC4* in a patient who underwent SNP-array analysis for intellectual disability and dysmorphisms.

### Case reports

#### CCM067

**Figure 1:**
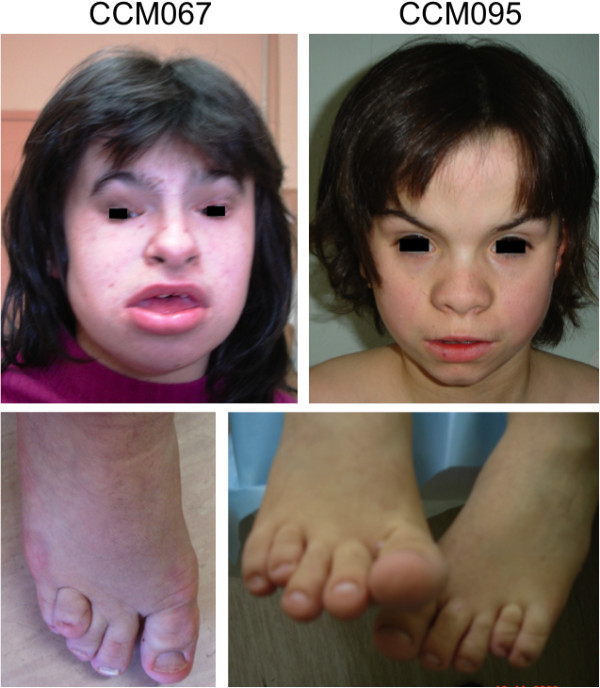
**Faces and toe dysmorphisms from patients CCM067 and CCM095.** Note the distinct coarse face with unusually shaped eyebrows (arched or upsweeping, full or thick, with synophrys), wide and prominent nasal tip, full and everted lower lip, and the digital anomalies with recessed 4th toes.

**Figure 2:**
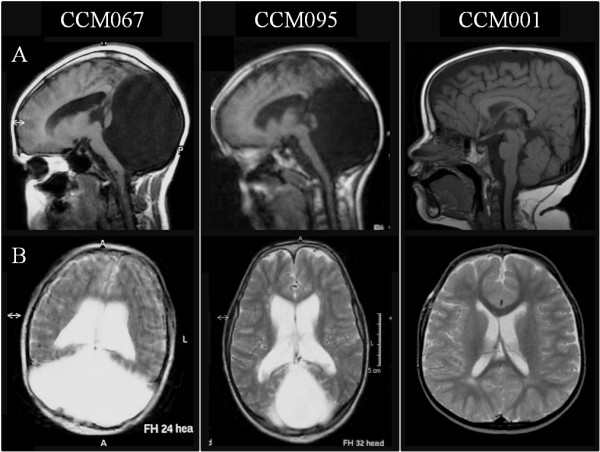
**Brain MRIs.** Brain imaging of patients CCM067, CCM095 (with Dandy-Walker malformation) and CCM001 (with normal cerebellum and posterior fossa). **A**: sagittal midline T1-weighted images. **B**: axial T2-weighted images.

This 21-year old Macedonian girl is the first child of non-consanguineous parents. Family history, pregnancy and delivery were unremarkable. Birth weight and length were at the 50th and 75th centile, respectively. Soon after birth, the child showed severe hypotonia, with poor sucking and no crying. An umbilical hernia was diagnosed at birth. At age 3 months, a ventricular derivation was placed to treat hydrocephalus. There was global psychomotor delay (sitting at 13 months, walking and first words without structured language at 3.5 years), and severe intellectual disability, with scholarship impossible. Clinical examination at 17 years revealed dysmorphic features including blepharophimosis, ptosis and epicanthus inversus, upslanting palpebral fissures, posteriorly rotated ears and coarse facial features, with unusually shaped bushy eyebrows, synophrys, broad and prominent nose, short simplified philtrum, macrostomia with full lower lip, macroglossia, and broad neck. The patient also presented short stature (140 cm, <3rd centile), axillary freckles, hypoplastic nipples, scoliosis and peculiar toe anomalies. These included recessed and overriding right 4th toe, shown by X-ray to be related to short IV metatarsal, and broad halluces with sandal gap (Figure 1). Neurological examination showed spasticity and gait ataxia. Severe intellectual disability was confirmed by Griffith’s testing. Brain magnetic resonance imaging (MRI) at 17 years showed a typical DWM, with a small, rotated remnant of the vermis and a very large posterior fossa cyst. There were also enlarged lateral ventricles, thin corpus callosum and severe pons hypoplasia (Figure 2).

#### CCM095

This is a 11-year old Macedonian girl, born to non-consanguineous parents. Pregnancy was normal and delivery at term by cesarean section. Birth weight and length were at the 50th centile. The patient suffered from neonatal asphyxia, generalized hypotonia and poor sucking, with Apgar scores 2^1′^ and 5^5′^. Club foot, umbilical and inguinal hernias were also noted at birth. Developmental milestones were grossly retarded with head control at 2 years, sitting at 2.5 years, walking and first words at 4 years. She never acquired structured language, and communication was possible only with gestures. Clinical examination revealed frontal bossing, low-set floppy ears, coarse facial features with long arched eyebrows, broad and bulbous nose, full lower lip, and mild micrognathia. Skeletal anomalies were also present, including asymmetric thorax with pectus excavatum, kyphoscoliosis, hand brachydactyly, broad halluces and recessed 4th toes (Figure 1). Neurological examination disclosed spasticity with bilateral ankle clonus and Babinski sign. A history of epilepsy was also reported. The patient had severe intellectual disability and scholarship was impossible. Precocious puberty occurred at age 5.5 years (PH2B2 Tanner stage) with elevated FSH and LH levels, and an advanced bone age of about 11 years; for this she was treated with Decapeptyl until age of 10 years. Brain MRI at 9 years revealed DWM with a severely hypoplastic and rotated cerebellar vermis, large posterior fossa cyst, severe pons and corpus callosum hypoplasia and hydrocephalus (Figure 2).

#### CCM001

This 5 year old girl was born to non-consanguineous Italian parents. Delivery was at term by cesarean section due to podalic presentation, after a pregnancy complicated by placental abruption. Apgar scores were 8^1′^ and 8^5′^. Birth length was 45 cm (<3rd centile), while weight and head circumference were normal. At birth she presented with neonatal hydronephrosis and atrial septal defect, which subsequently closed spontaneously. Oxygen therapy was introduced for desaturation in the first days of life. A neurological assessment at age 3 months showed generalized hypotonia, reduced motility and a mild head lag at the pull-to-sit test. Despite the negative newborn screening results, a congenital hypothyroidism was reported with mildly increased TSH levels, which required levothyroxine treatment. Maternal hypothyroidism was also reported. There was global delay of developmental milestones (rolling at 9 months, sitting at 18 months, first bisyllabic words at 26 months). When last examined at 5 years, the patient was unable to walk unsupported and presented gait ataxia, generalized hypotonia and speech impairment. She showed brachycephaly, frontal bossing, sparse eyebrows with mild synophrys, upslanting palpebral fissures, low set and posteriorly rotated ears, “puffy” cheeks, midfacial hypoplasia, mild micrognathia and bilateral single transverse palmar crease. Height was always below the 3rd centile, while weight fell at the 3rd centile since the second year. Brain MRI at age 2 years was unremarkable, with only mildly enlarged frontal subarachnoid spaces and subtle white matter hyperintensities of unclear significance. The cerebellum and other posterior fossa structures were normal (Figure 2).

### Genetic results

The three patients described here were found to carry overlapping deletions of the long arm of chromosome 3. In patient CCM067, the deletion spanned about 20 Mb in 3q22.3q25.31, from 136842221 bp (CN_984754) to 156872233 bp (CN_991783). Patient CCM095 also had a large deletion of about 20 Mb at 3q23q26.1, from 141846105 bp (CN_1010953) to 161983066 bp (SNP_A-8642959). Patient CCM001 showed two *de novo* deletions, a 4.9 Mb deletion at 3q24 locus from 142404332 bp (CN_978469) to 147269266 bp (CN_1006774), and a 3 Mb deletion at 11p11.2 locus from 45391614 bp (SNP_A-8698252) to 48636415 bp (CN_570217), partially overlapping the Potocki-Shaffer syndrome (PSS; MIM #601224) critical region. In all three patients, the 3q deletions included both *ZIC1* and *ZIC4* genes.

We performed SNP array analysis also in a 3q deleted patient with DWM and blepharophimosis, ptosis, and epicanthus inversus syndrome (BPES), whose detailed clinical features have been recently published [8]. This patient was found to carry a 19.2 Mb deletion of the region 3q22.1q25.1, from 132381260 bp (CN_995516) to 151675126 bp (CN_998137), including the *FOXL2*, *ZIC1* and *ZIC4* genes.

**Figure 3:**
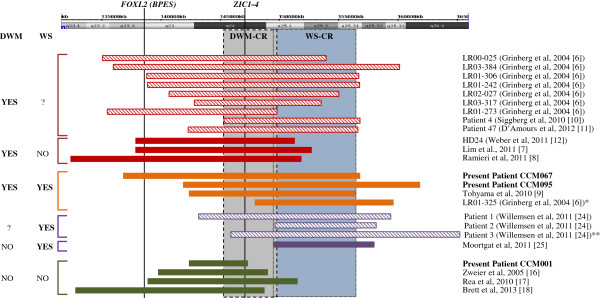
**Schematic representation of overlapping 3q deletions in DWM and WS patients.** Extension of 3q deletions in the present three and 21 previously published subjects (only patients with molecular characterization have been included). Vertical grey and blue areas represent the proposed critical regions for DWM and WS, respectively. Horizontal bars are grouped by color according to the presence/absence of either condition, as specified in the columns on the left. Red and purple shaded bars represent patients with unknown status for WS or DWM, respectively. Vertical black lines indicate the position of *FOXL2* gene (causative of BPES), and *ZIC1-ZIC4* genes, as indicated. *previously described by Sudha et al. [26]; **previously described by Ko et al. [23].

A graphical representation of the identified deletions plotted against chromosome 3q map is presented in Figure 3.

## Discussion

The genetic basis of DWM is complex and the involved genes are still largely unknown. The identification of seven DWM patients with overlapping deletions at 3q first implicated the *ZIC1* and *ZIC4* genes as causative of the malformation [6]. These genes encode for zinc finger transcription factors homologs of Drosophila melanogaster odd-paired genes, and are widely expressed in the dorsal central nervous system, including the developing cerebellum and spinal cord. Doubled *Zic1+/− Zic4+/−* heterozygous mice display a mild to severe cerebellar phenotype, with foliar defects and disproportionate hypoplasia of the vermis compared to the hemispheres, mimicking the cerebellar morphology of human DWM [6]. A recent study demonstrated that *Zic1* and *Zic4* have both a Shh-dependent and independent function, promoting proliferation of granule cell progenitors and regulating expression of genes involved in cerebellar anlage patterning and vermis foliation [15].

Here we report two novel patients (CCM067 and CCM095) with a severe DWM phenotype and large deletions encompassing both *ZIC1* and *ZIC4* genes. Furthermore, six additional DWM patients with 3q deletions have been published since the original report [7-12], defining a critical region clearly implicated in DWM pathogenesis (DWM-CR in Figure 3). In a patient (LR01-325) [6], whose deletion did not encompass *ZIC1-ZIC4*, their expression levels were significantly reduced compared to controls, implying a position effect, possibly exerted by distally located regulatory elements.

On the other hand, some evidence suggests that *ZIC1* and *ZIC4* haploinsufficiency is neither necessary nor sufficient *per se* to cause DWM. In fact, we failed to identify deletions of these genes in 11 patients with a diagnosis of isolated or syndromic DWM. Furthermore, we report here on a patient (CCM001), heterozygous for a small 3q24 deletion encompassing both *ZIC1*-*ZIC4* genes, who did not display any of the neuroradiological features defining the DWM spectrum. By reviewing the clinical data of well characterized individuals with deletions encompassing the *ZIC1* and *ZIC4* genes, we found three additional patients lacking any cerebellar or posterior fossa anomalies at ultrasound or CT scan [16-18]. Noticeably, the telomeric boundaries of 3q deletions map well beyond the DWM-CR in several DWM patients, suggesting that another locus, distal to *ZIC1* and *ZIC4*, could contribute with a possible additive effect to DWM pathogenesis.

Patient CCM001 also showed a *de novo* deletion at 11p11.2 of about 3 Mb. The deletion partially overlaps to the critical region of PSS, characterized by developmental delay and intellectual disability, hypotonia, craniofacial and ophthalmologic anomalies, multiple exostoses and parietal foramina [19]. In our patient, the lack of exostoses and parietal foramina is in agreement with the presence of two copies of the causative genes *EXT2* and *ALX4*. Conversely, the deletion includes the *PHF21A* gene, which has been recently implicated as contributing to the intellectual disability and craniofacial anomalies typical of PSS, such as brachycephaly, midfacial and mandibular hypoplasia [20].

Facial dysmorphisms in our patient CCM067 are characteristic of BPES, consistent with her 3q deletion encompassing *FOXL2*, the BPES causative gene [21]. However, this patient also had additional distinctive features, including upslanting palpebral fissures, high arched bushy eyebrows, coarse facies, prominent nose, large mouth, full lower lip, and a peculiar short IV metatarsus. Intriguingly, patient CCM095 also had a similar phenotype. Taken together, these features were highly reminiscent of Wisconsin syndrome (WS), a condition first described in 2000 by Cohen based on a patient seen in 1976 by Opitz [22], and then confirmed in another patient carrying a 3q deletion [23].

Three subjects with 3q deletions and the WS phenotype, including the patient described by Ko et al. [23], have been recently re-evaluated by microarray analysis, mapping the critical region to chromosome 3q24q25 [24]. Indeed, our patients CCM067 and CCM095 corroborated a relationship between WS and interstitial 3q deletions, prompting us to perform a detailed assessment of the available photographs and clinical features of published cases with deletions encompassing 3q24 and/or 3q25 (Additional file 1: Table S1). Based on this analysis, we propose the diagnosis of WS to be made based on the occurrence of at least four out of five core gestaltic features (coarse facies; prominent or wide triangular shaped nasal tip; high arched or upsweeping eyebrows; full/everted lower lip; bushy eyebrows often with synophrys). Accordingly, we diagnosed 12 patients with WS [6,9,22,23,25-29] and compared them with 15 patients who did not match the proposed criteria [7,8,12,16-18,30-37] (Table 1 and Additional file 1: Table S1). Among this second group, the occurrence of each gestaltic feature was much rarer than in the WS group. Moreover, we identified additional features frequently observed in WS patients. Some of these, such as intellectual disability, smooth philtrum and ear anomalies, were found at similar frequencies also in non-WS patients, while others appeared to be more specific of the WS phenotype. In particular, digital anomalies were described in ten out of 12 WS patients, of whom four presented a peculiar brachydactyly of the 4th toe (see Figure 1). Conversely, some features reported in the original WS patients, such as craniosynostosis [22] or hypogonadism [23,24], do not appear to be common features in WS.

**Table 1:** Comparison of selected clinical features in WS vs non-WS patients with 3q deletions

	WS (n = 12)*	Non-WS (n = 15)*
**Core gestaltic features**		
Coarse facies	12 (100%)	0
Prominent or wide triangular shaped nasal tip	12 (100%)	3 (20%)
High arched or upsweeping eyebrows	11 (92%)	0
Full/everted lower lip	11 (92%)	5 (33%)
Bushy eyebrows	10 (83%)	3 (20%)
**Other recurrent clinical features**		
Developmental delay/intellectual disability	12 (100%)	15 (100%)
Digital anomalies:	10 (83%)	6 (40%)
-of which short IV metatarsus	4 (33%)	0
Ear anomalies	9 (75%)	13 (87%)
Macrostomia	8 (67%)	1 (7%)
Smooth/simplified philtrum	7 (58%)	7 (47%)

*Identification codes, detailed clinical features, cytogenetic characterization and references for patients with and without WS are given in Additional file 1: Table S1.

A molecular characterization of the deletions’ breakpoints was available for eight of the 12 WS patients [6,9,24,25], allowing to define a critical region of 7 Mb in 3q25 (WS-CR, Figure 3). This region contains 43 RefSeq genes, none of which appears as a strong candidate for WS.

## Conclusions

Our findings indicate that the deletion pattern in chromosome 3q is more complex than previously suggested, resulting in three distinct phenotypes that depend on the extension of the rearrangement. Only deletions extending proximally to 3q22.3, encompassing *FOXL2* gene, are associated with features of BPES (as in our CCM067 case). Deletions of 3q24 region, including *ZIC1* and *ZIC4* genes, can be associated to DWM, but penetrance is incomplete. Finally, interstitial deletions extending telomeric to this region, involving 3q25 band, could lead to the WS phenotype. Brain MRI should be warranted in all these patients, to assess the presence of cerebellar and brainstem malformations.

## Abbreviations

bp: base pairs; BPES: Blepharophimosis, ptosis, and epicanthus inversus syndrome; CNV: Copy number variation; CR: Critical region; DWM: Dandy-Walker malformation; Mb: Megabases; MRI: Magnetic resonance imaging; PF: Posterior fossa; PSS: Potocki-Shaffer Syndrome; WS: Wisconsin Syndrome.

## Competing interests

The authors declare that they have no competing interests.

## Authors’ contributions

Patients’ recruitment and analysis of clinical and imaging data: AF, LB, FM, EMV; SNP-array studies: LB, SL, VP, AC, AN, SB; patients referral and clinical data collection: VS-A, GZ, ES-A, ST, LT, FD, EM, LT, EB, BD; literature review and dysmorphological evaluation: AF, BD; study conception and design, manuscript drafting: AF, LB, EMV. All authors read and approved the final manuscript.

## Supplementary Material

Additional file 1: Table S1 Detailed clinical features in WS versus non-WS patients with 3q deletions. This table includes only published patients with pure deletions encompassing 3q24 and/or 3q25, with available clinical descriptions and/or pictures. Data from 12 WS and 15 non-WS patients are listed in two separate spreadsheets. Click here for file
